# Management of low-risk papillary thyroid cancer. Minimally-invasive treatments dictate a further paradigm shift?

**DOI:** 10.1007/s12020-024-03864-7

**Published:** 2024-05-20

**Authors:** E. Papini, R. Guglielmi, R. Novizio, A. Pontecorvi, C. Durante

**Affiliations:** 1 Department of Endocrinology and Metabolism, Regina Apostolorum Hospital, Albano, Rome, Italy; 2 Department of Endocrinology, Catholic University of The Sacred Heart, Policlinico Universitario “A. Gemelli” IRCCS, Rome, Italy; 3 Department of Translational and Precision Medicine, Sapienza University of Rome, Rome, Italy

**Keywords:** Papillary thyroid cancer, Minimally invasive procedures, Thermal ablation, Laser, Radiofrequency, Microwaves

## Abstract

**Background:**

Current management options for PTMC include lobo-isthmectomy and active surveillance (AS). Recently, ultrasound-guided minimally invasive procedures (MITs) are offered as a nonsurgical therapy for PTMC because they do not require hospitalization and general anaesthesia, and do not result in loss of thyroid function or cosmetic damage. MITs are reported to consistently provide, mostly in large retrospective series of patients, a rapid, safe, and cost-effective way to eradicate low-risk thyroid malignancies. However, conclusive data from well-conducted prospective studies on the histologically-proven completeness of tumor ablation and the long-term clinical advantages versus AS are still lacking.

**Objectives:**

This study aimed to evaluate the efficacy and safety of ultrasound-guided minimally invasive treatments (MITs) for PTMC in comparison to traditional surgical methods and active surveillance, and to assess their role in current clinical practice.

**Methods:**

A structured literature review was conducted using keywords related to PTMC, MIT, and comparative techniques. Studies were evaluated based on treatment modality, patient selection, follow-up duration, complication rates, and clinical outcomes.

**Results:**

MITs have shown promising results in the management of PTMC. These treatments offer several advantages over surgery, such as reduced use of surgical resources, lower costs, minimal work disruption, and fewer major complications. However, there are still limitations, including the need for long-term surveillance and the potential risk of incomplete tumor ablation.

**Conclusions:**

MITs represent a promising non-surgical option for managing low-risk PTMC, especially for patients ineligible for or refusing surgery. Despite favorable outcomes, more robust prospective data are needed to confirm their long-term benefits and completeness of tumor ablation. Interdisciplinary discussions and thorough patient education on the advantages and limitations of MITs are crucial for informed decision-making.

## Background

The diagnosis of papillary thyroid carcinoma (PTC) has become more frequent during the last decades due to the widespread availability of neck imaging techniques, routine use of ultrasound (US)-guided fine needle aspiration biopsy (FNA), and, presumably, to the actual increase in the incidence of differentiated thyroid tumors [1]. Almost half of these cancers are under 10 mm in size and slow-growing, often incidental, and associated with a favorable prognosis. The majority of these tumors are low-risk papillary thyroid carcinomas (PTC), lacking aggressive histological features, extrathyroidal spread, nodal or distant metastasis, and worrisome mutations. These very low-risk cancers are generally referred to as papillary thyroid microcarcinomas (PTMC) in the literature, although the 2022 WHO classification does not consider them as distinct pathological conditions [2].

Currently, there is robust evidence supporting a minimalistic approach to the management of PTMC. The recommended surgical treatment for unifocal PTMC is lobo-isthmectomy but active surveillance (AS) is increasingly being proposed as an alternative management option. Several long-term studies, conducted in various thyroid centers across different countries, have demonstrated the safety of clinical and US monitoring without immediate intervention [3–7]. Recently, a third management option has been proposed to prevent the risk of overtreatment of clinically insignificant tumors and is now being tested worldwide [8]. Minimally invasive treatments (MIT), performed under US guidance, are established as effective and safe therapeutic tools for symptomatic benign thyroid nodules [9–12]. MITs are now being considered also as a possible nonsurgical therapy for PTMC because they do not require hospitalization, general anaesthesia, and do not result in loss of thyroid function or cosmetic damage [9, 11–13]. Over the last 10 years, several clinical studies have shown that thermal ablation procedures—performed either with laser (LTA), radiofrequency (RFA), or microwaves (MWA) devices—can be used to selectively ablate PTMC with a diameter of up to 10 mm. Thus, the choice of the most appropriate management—surveillance, thermal ablation, or surgery—for these typically indolent tumors necessitates a thorough assessment of individual needs, clinical circumstances, and preferences.

Aim of the present paper is to assess the level of evidence for the outcomes of MITs in the treatment of PTMC, in order to evaluate the advantages and limitations of these innovative techniques in comparison to surgery and AS and to define their present role in current clinical practice.

## Methods

To conduct the literature review on the management of Papillary Thyroid Microcarcinoma (PTMC), especially focusing on minimally invasive treatments, a structured search strategy using both specific keywords and Boolean operators has been employed. Keywords such as “PTMC”, “Papillary Thyroid Microcarcinoma”, “Thermal ablation”, “Laser”, “Radiofrequency”, “Microwave”, “Active Surveillance” guided the initial search. The search included Boolean combinations such as (“Papillary Thyroid Microcarcinoma” OR “PTMC” OR “Low-risk Papillary Thyroid Carcinoma”) AND (“minimally invasive procedures” OR “thermal ablation” OR “minimally invasive treatment” OR “ablation” OR “ablat*”) and Thyroid NEAR/3 Ablation to focus on articles discussing specific techniques used in the minimally invasive approach. Additionally, the comparison between surgical and non-surgical techniques has been explored using the string “Thyroid Surgery” VS “Non-Surgical Techniques” AND “PTMC”, allowing for a focused retrieval of studies comparing these approaches. For broader technology-based searches, (“Papillary Thyroid Microcarcinoma” OR “PTMC” OR “PTCM” OR “Papillary Thyroid Carcinoma”) AND (“laser” OR “LTA” OR “LT” OR “radiofrequency” OR “RFA” OR “microwaves” OR “MWA”) has been used to include various technologies applied in PTMC treatments. To investigate the long-term efficacy and patient-centered outcomes, strings such as “Active Surveillance” AND (“Papillary Thyroid Microcarcinoma” OR “PTMC” OR “PTCM” OR “Papillary Thyroid Carcinoma”) AND “outcomes” and (“Cost-Effectiveness” OR “Quality of Life”) AND (“Thermal Ablation” OR “Minimally Invasive”) AND “Thyroid Cancer”.

## Available evidence

After the initial feasibility studies and pilot trials [14–16], several studies, performed with various minimally invasive technologies, investigated the efficacy, safety, and long-term outcomes of these treatment. Table 1 includes the main studies on the use of MIT for low-risk PTMC and summarizes modalities of treatment, patients’ selection, follow-up duration, complications rate, and clinical outcomes.

**Table 1:** Main studies on the use of MIT for low-risk PTC

Study	Study design	Energy source	Pt. (Tumor) [Surgery]	Comparison vs surgery [Type of surgery]	FU months [Surgery]	Local persistence/recurrence [Surgery]	New tumor [Surgery]	Distant mts [Surgery]	LN mts [Surgery]	Rescue surgery [Surgery]
Zhou et al. [16]	Retro.	LTA	30 (30)	No	24	0	0	0	0	0
Xu et al. [27]	Retro.	MWA	41 (41) [46 (46)]	Yes [Lobectomy + CND]	1 [1]	0 [0]	0 [0]	0 [0]	0 [0]	0 [0]
Teng et al. [28]	Pros.	MWA	15 (21)	No	36–48	0	0	0	0	0
Teng et al. [29]	Pros.	MWA	185 (206)	No	20.7 ± 8.8	0	1 (0.5%)	0	0	0
Li et al. [30]	Retro.	MWA	168 (168) [143 (143)]	Yes [Lobectomy + CND]	25.1 [27.5]	0 [0]	2 [1]	0 [0]	5 [5]	2 [2]
Zhou et al. [31]	Retro.	LA	36 (36) [45 (45)]	Yes [Lobectomy + CND]	49.2 [48.5]	0 [0]	1 [1]	0 [0]	1 [0]	2 [1]
Wang et al. [32]	Pros.	/	107 (107)	No	18	0	0	0	0	0
Zhang et al. [33]	Retro.	RFA	94 (94) [80 (86)]	Yes [Lobectomy 19 pts] [Lobectomy + CND, 39 pts] [TT 8 pts] [TT + CND 14 pts]	64.2 [63.6]	0 [0]	1 [1]	0 [0]	0 [1]	0 [/]
Yue et al. [34]	Pros.	MWA	119 (119)	No	37.2 ± 20.9	0	0	0	1	0
Cho et al. [35]	Pros.	RFA	74 (84)	No	72	0	0	0	0	0
Xiao et al. [36]^a^	Retro.	RFA	66 (66)	No	20.5 ± 7.4	2 (3.0%)	0	1 (1.5%)	0	0
Cao et al. [37]^b^	Retro.	RFA MWA	673	No	21 ± 12	0	5 (0.7)	0	1 (0.1)	1 (0.1)
Cao et al. [37]^c^	Retro.	RFA MWA	174	No	25 ± 14	0	2 (1.1)	0	1 (0.6)	1 (0.6)
Cao et al. [38]	Retro.	RFA MWA	725 (725)	No	6	0	5 (0.7)	0	1 (0.1)	/
Wang et al. [39]^d^	Retro.	MWA	63 (63) [83 (83)]	Yes [TT + lymph node dissection on the affected side]	24	0 [0]	0 [1 (1.2%)]	0 [0]	0 [1 (1.2%)]	0 [0]
Xiao et al. [40]^e^	Retro.	RFA	73 (73)	No	24.1 ± 6.9	0	0	0	0	0
Zheng et al. [41]^f^	Retro.	MWA	21 (21)	No	32	0	0	0	0	0
Li et al. [42]	Pros.	RFA MWA	30 (30) [31 (31)]	Yes [Lobectomy 21 pts] [Lobectomy + CND, 7 pts] [TT 2 pts] [TT + CND 1 pts]	16.4 ± 5.2 [/]	0 [/]	0 [/]	0 [/]	0 [/]	0 [/]
Wu et al. [43]^g^	Retro.	MWA	106 (106)	No	42	0	2	0	2	7
Mauri et al. [44]	Pros.	LTA (3) RFA (8)	11 (11)	No	10.2	0	0	0	0	0
Yan et al. [45]^h^	Retro.	RFA	332 (332) [332 (332)]	Yes [Lobectomy pts]	48.3	1, 0.3% [0, 0%]	8, 2.4% [4, 1.2%]	0 [0]	2, 0.6% [2, 0.6%]	0 /
Yong-Ping et al. [46]	Retro.	LTA	43 (43)	No	23.47 ± 6.5	0	1 (2.3%)	0	0	/
Zhang et al. [47]^i^	Pros.	LTA	12 (26)	No	19.75 ± 11.46	0	1 (8.3%)	0	0	0
Zhang et al. [47]	Pros.	LTA	60 (60)	No	16.33 + 10.01	0	3 (5%)	0	0	0
Zhao et al. [48]	Retro.	MWA	341 (341)	No	24	0	0	0	0	0
Zheng et al. [49]^j^	Pros.	MWA	92 (92) [196 (106)]	Yes [Lobectomy + CND] [Loboisthmectomy + CND] [TT + CND]	12.7 ± 4.1 [12.6 ± 5.0]	/	/	/	/	/
Yan et al. [50]	Retro.	RFA MWA	RFA 375 (375) MWA 117 (117)	No	77.2	17 (3.6%)	12 (2.5%)	0	5 (1.1%)	/
Han et al. [51]	Pros.	MWA	1278 (1278)	No	34.57 ± 28.98	6 (0.46%)	0	0	10 (0.78%)	4
Li et al. [26]^b,k^	Retro	RFA	341 (341)	No	67.8 ± 18.2	10 (2.9%)	0	0	/	0
Li et al. [26]^c,k^	Retro	RFA	41 (41)	No	67.8 ± 18.2	5 (12.2%)	0	0	/	0

Modalities of treatment, patients’ selection, follow-up duration, modalities of assessment of ablation effectiveness, complications, and outcomes are summarized

/ - Not reported or available

^a^Only pT1b PTC were included in this study

^b^This study considered both T1a and T1b. In this line, only T1a data are considered

^c^This study considered both T1a and T1b. In this line, only T1b data are considered

^d^Only PTC < 6 mm have been included in this study

^e^Only pT1b PTC were included in this study

^f^This study evaluated isthmic PTMC only

^g^This study included T1a and T1b close to the thyroid capsule

^h^460 pts were included in Lobectomy group and 424 were included in RFA group before propensity score matching was applied

^i^This study evaluated mutifocal PTMC

^j^This study included PTCs with sonographically detected mETE

^k^1 metastatic lymph node has been reported during follow-up, not specifying if in T1a or T1b group

### Advantages of minimally invasive treatments versus surgery

The benefits of thermal ablation in the management of low-risk PTMC are potentially relevant and encourage considering them as an alternative to surgery in selected conditions:

- No use of surgical resources. Reducing the number of thyroid surgeries for low-risk PTMC could led to more appropriate allocation of resources and time towards surgery for patients with advanced thyroid cancer or large goiter that cause local pressure symptoms [9–12].
- Low cost of the procedure. Due to the absence of general anaesthesia, surgical staff employment, and in-hospital stay, a relevant decrease of the costs may be achieved in comparison to thyroidectomy [17, 18].
- Minimal loss of working days. The persistence of local symptoms is generally limited to 24–48 h and the general clinical conditions and working ability are only minimally affected by MIT [18].
- Low risk of major complications. Lower occurrence of peri-operatory complications and of permanent dysphonia are consistently reported in comparison to surgery [19].
- Loss of thyroid function. Replacement therapy is only anecdotally required after MIT while is necessary, over time, in a sizable number of patients treated with lobectomy [19, 20].
- Absence of cosmetic damage. Cervical scars are avoided without the need for complex, and more expensive, trans-axillary or trans-oral surgical techniques [18, 20–22].

### Advantages of minimally invasive treatments versus AS

A few advantages can be attributed to MITs due to the following considerations:

- A linear growth of PTMC volume may occur in about 8% of AS patients and may eventually prompt a delayed surgical treatment [6, 23].
- A minority of patients, especially young subjects, may develop over time cervical lymph node metastases that could require a more extensive, and potentially more disfiguring, neck surgery [23].
- A substantial number of patients in AS programs eventually undergo thyroidectomy for reasons unrelated to cancer growth, mostly anxiety due to the awareness of harboring an untreated malignancy [24].
- It can be speculated that only a limited number of centers can offer a reliable life-long clinical and US surveillance for a steadily increasing number of patients.

### Grey zones

A conclusive definition of the role of MITs as a first line therapeutic option in the management of PTMC still has limitations that should be specifically addressed in future trials:

- *Completeness of tumor ablation*. The histological confirmation of the complete ablation of malignant tissue is based on few anecdotal cases and on minute series of patients who, for unrelated reasons, underwent thyroidectomy after MITs [15, 25]. Only few papers report the regular use of FNA or core needle biopsy (CNB) for ruling out the persistence of viable tumor cells in the treated area (Table 2). A retrospective cohort study, performed with long-term US follow up and systematic assessment with CNB of the ablation zone provided reassuring information [26]. Conversely, the risk of an incomplete ablation cannot be excluded with certainty in the majority of MIT studies which used US evaluation alone for the clinical outcome assessment. This issue is of pivotal importance because the eradication of any viable tumor cell represents the major advantage of MITs versus AS in the management of PTMC. The challenges of achieving complete tumor ablation are further compounded by the following factors. First, nearly all the available series include a relevant number of very small size (5 mm or less in diameter) PTMC (Table 3). Due to the need of a 2 mm circumferential safety margin of ablation around the PTMC circumference [8] the risk of an oncologically incomplete ablation rapidly increases with the increment in tumor size and is, consequently, much greater for tumors close to 10 mm. Second, the location of PTMC in areas difficult to be treated—close to the trachea, major vessels, or laryngeal nerve course—may hamper the radicality of the ablation even after a well conducted hydrodissection. Third, in case of incomplete ablation, the sonographic changes induced by MIT might hinder the persistence or recurrence of the treated PTMC.
- *Risk of complications*. Head-to-head randomized prospective studies comparing MITs vs lobectomy are lacking. The current evidence is mostly based on retrospective studies comparing two cohorts of patients after non-randomized enrolment (Table 4). Besides this methodological limitation, most available studies compare thermal ablation, performed with different technologies and variable modalities, to surgery performed with lobectomy, total thyroidectomy, or thyroidectomy with central compartment resection. Differences in complications are most significant when comparing MITs to the most extensive surgical options, favoring MITs, but diminish when compared to lobectomy, the current recommended choice for managing unifocal carcinomas.
- *Quality of life*. Surgical interventions are expected to negatively affect quality of life. and a better tolerability of MIT procedures may be postulated. Yet, controlled studies comparing the peri-operative and long-term impact on the QoL of the three management alternatives with validated and internationally accepted questionnaires are scarce.
- *Long-term surveillance*. Surgery rules out the risk of tumor recurrence in the affected lobe and provides accurate information about tumor multifocality, extrathyroidal extension, aggressive histology, or clinically significant lymph node involvement. If these data, which conclusively define the risk of recurrence, are lacking, a prolonged though not intensive clinical and sonographic follow-up is required after thermal ablation, similarly to AS.
- *Access to treatment*: a limited number of centers currently offer MIT for treating PTMC and specific training courses and certifications are not available in most countries.

**Table 2:** Thermally ablated PTC followed up through core needle biopsy

Study	Study design	Energy source	Pt. (Tumor) [Surgery]	Comparison vs surgery [Type of surgery]	FU months [Surgery]	Local persistence/recurrence [Surgery]	New tumor [Surgery]	Distant mts [Surgery]	LN mts [Surgery]	Rescue surgery [Surgery]
Xiao et al. [36]^a^	Retro.	RFA	66 (66)	No	20.5 ± 7.4	2 (3.0%)	0	1 (1.5%)	0	0
Li et al. [26]^b,c^	Retro	RFA	341 (341)	No	67.8 ± 18.2	10 (2.9%)	0	0	/	0
Li et al. [26]^c,d^	Retro	RFA	41 (41)	No	67.8 ± 18.2	5 (12.2%)	0	0	/	0

/ - Not reported or available

^a^Only pT1b PTC were included in this study

^b^This study considered both T1a and T1b. In this line, only T1a data are considered

^c^1 metastatic lymph node has been reported during follow-up, not specifying if in T1a or T1b group

^d^This study considered both T1a and T1b. In this line, only T1b data are considered

**Table 3:** Only PTC < 6 mm have been included in this study

Study	Study design	Energy source	Pt. (Tumor) [Surgery]	Comparison vs surgery [Type of surgery]	FU months [Surgery]	Local persistence/recurrence [Surgery]	New Tumor [Surgery]	Distant mts [Surgery]	LN mts [Surgery]	Rescue surgery [Surgery]
Wang et al. [39]	Retro.	MWA	63 (63) [83 (83)]	Yes [TT + lymph node dissection on the affected side]	24	0 [0]	0 [1 (1.2%)]	0 [0]	0 [1 (1.2%)]	0 [0]

**Table 4:** Retrospective studies comparing MITs vs lobectomy

Study	Study design	Energy source	Pt. (Tumor) [Surgery]	Comparison vs surgery [Type of surgery]	FU months [Surgery]	Local persistence/recurrence [Surgery]	New tumor [Surgery]	Distant mts [Surgery]	LN mts [Surgery]	Rescue surgery [Surgery]
Xu et al. [27]	Retro.	MWA	41 (41) [46 (46)]	Yes [Lobectomy + CND]	1 [1]	0 [0]	0 [0]	0 [0]	0 [0]	0 [0]
Li et al. [30]	Retro.	MWA	168 (168) [143 (143)]	Yes [Lobectomy + CND]	25.1 [27.5]	0 [0]	2 [1]	0 [0]	5 [5]	2 [2]
Zhou et al.[31]	Retro.	LA	36 (36) [45 (45)]	Yes [Lobectomy + CND]	49.2 [48.5]	0 [0]	1 [1]	0 [0]	1 [0]	2 [1]
Zhang et al. [33]	Retro.	RFA	94 (94) [80 (86)]	Yes [Lobectomy 19 pts] [Lobectomy + CND, 39 pts] [TT 8 pts] [TT + CND 14 pts]	64.2 [63.6]	0 [0]	1 [1]	0 [0]	0 [1]	0 [/]
Wang et al. [39]^a^	Retro.	MWA	63 (63) [83 (83)]	Yes [TT + lymph node dissection on the affected side]	24	0 [0]	0 [1 (1.2%)]	0 [0]	0 [1 (1.2%)]	0 [0]
Li et al. [42]	Pros.	RFA MWA	30 (30) [31 (31)]	Yes [Lobectomy 21 pts] [Lobectomy + CND, 7 pts] [TT 2 pts] [TT + CND 1 pts]	16.4 ± 5.2 [/]	0 [/]	0 [/]	0 [/]	0 [/]	0 [/]
Yan et al. [45]^b^	Retro.	RFA	332 (332) [332 (332)]	Yes [Lobectomy pts]	48.3	1, 0.3% [0, 0%]	8, 2.4% [4, 1.2%]	0 [0]	2, 0.6% [2, 0.6%]	0 /
Zheng et al. [51]^c^	Pros.	MWA	92 (92) [196 (106)]	Yes [Lobectomy+CND] [Loboisthmectomy + CND] [TT + CND]	12.7 ± 4.1 [12.6 ± 5.0]	/	/	/	/	/

/ - Not reported or available

^a^460 pts were included in Lobectomy group and 424 were included in RFA group before propensity score matching was applied (i)

^b^Only PTC < 6 mm have been included in this study (e)

^c^This study included PTCs with sonographically detected mETE (j)

## Conclusions for clinical practice

Thermal ablation is a promising approach for non-surgical management of low-risk PTMC. Minimally invasive treatments could provide a rapid, safe, and cost-effective way to eradicate these common malignancies. However, conclusive data from well-conducted prospective studies on the histologically-proven completeness of tumor ablation and the long-term clinical advantages versus active surveillance are still lacking.

Presently, MIT should be considered in high-volume thyroid centers for patients with PTMC who are not candidates for surgery or refuse it, but still seek treatment to decrease the risk of progressive growth or extrathyroidal spread of their malignancy over time.

The three available therapeutic options—lobectomy, thermal ablation, and active surveillance—should always be discussed in an interdisciplinary manner. This discussion should be based not only on the preliminary clinical and US staging but also on the patient’s preferences, available resources, and local expertise.

Patients undergoing MIT for PTMC should be fully informed about the advantages and limitations of the procedure. Due to the presently incomplete level of evidence, long-term clinical and US follow-up is still required.
